# Self-Supervised Chinese Ontology Learning from Online Encyclopedias

**DOI:** 10.1155/2014/848631

**Published:** 2014-03-13

**Authors:** Fanghuai Hu, Zhiqing Shao, Tong Ruan

**Affiliations:** Department of Computer Science and Engineering, East China University of Science and Technology, Shanghai 200237, China

## Abstract

Constructing ontology manually is a time-consuming, error-prone, and tedious task. We present SSCO, a self-supervised learning based chinese ontology, which contains about 255 thousand concepts, 5 million entities, and 40 million facts. We explore the three largest online Chinese encyclopedias for ontology learning and describe how to transfer the structured knowledge in encyclopedias, including article titles, category labels, redirection pages, taxonomy systems, and InfoBox modules, into ontological form. In order to avoid the errors in encyclopedias and enrich the learnt ontology, we also apply some machine learning based methods. First, we proof that the self-supervised machine learning method is practicable in Chinese relation extraction (at least for synonymy and hyponymy) statistically and experimentally and train some self-supervised models (SVMs and CRFs) for synonymy extraction, concept-subconcept relation extraction, and concept-instance relation extraction; the advantages of our methods are that all training examples are automatically generated from the structural information of encyclopedias and a few general heuristic rules. Finally, we evaluate SSCO in two aspects, scale and precision; manual evaluation results show that the ontology has excellent precision, and high coverage is concluded by comparing SSCO with other famous ontologies and knowledge bases; the experiment results also indicate that the self-supervised models obviously enrich SSCO.

## 1. Introduction

An ontology is a formal description of a discourse domain. It typically consists of a finite list of concepts, the relationships (synonymous, taxonomic, and nontaxonomic) between these concepts, the instances of these concepts and the attributes of concepts, and instances. Ontologies are used widely in knowledge management, information retrieval, and intelligent recommendation. Moreover, ontologies are the basis of the next generation of the web, namely, the semantic web. However, constructing ontologies manually is very expensive, as it needs many domain experts and programmers to work together for a long time. Moreover, the manual process is tedious and error prone, and the ontologies built suffer from low coverage and fast aging. Thus, there are only a few manually built considerable ontologies; the most famous two are WordNet [1] and Cyc [2]. HowNet (http://www.keenage.com/) provides a knowledge database in Chinese, but it has fewer than 100,000 words and is not free.

In the last few years, researchers have employed themselves in constructing ontologies automatically or semi-automatically, which is called ontology learning. Depending on the coverage of the ontology, ontology learning can be divided into general ontology learning and domain ontology learning. When divided by from which ontologies are learnt, ontology learning can be based on structured data, semistructured data, and unstructured data; more concretely, semi-structured data include normal HTML pages, online encyclopedias search logs. With the development of Web 2.0, more and more collaboratively edited encyclopedic knowledge is being published online. Although the contents of these encyclopedias are not really machine interpretable, they contain much structured knowledge, such as redirection pages, category systems, and InfoBox modules, and are excellent corpora for ontology learning. The most famous online encyclopedia is Wikipedia, which contains more than 22 million articles in 285 different languages. Therefore, lots of ontology construction methods based on Wikipedia have been proposed [3–6], most of which are about English ontology construction as English-Wikipedia, which is the largest language edition of Wikipedia; all these methods have benefited from the abundant data and good structure of Wikipedia. When doing Chinese ontology construction, we find the following difficulties or differences.

- We do not have such good structured and large Chinese encyclopedias as the English Wikipedia: the Chinese-Wikipedia contains about half a million articles, only about one-seventh of that of English Wikipedia. Although we will make use of other two large Chinese encyclopedias, namely, Baidu-Baike (http://baike.baidu.com/, a Chinese language collaborative Web-based encyclopedia provided by the Chinese search engine Baidu.) and Hudong-Baike (http://www.hudong.com/, the world's largest Chinese encyclopedia.), each of which contains millions of articles, their sturctures are much worse for mapping into ontological form.
- We do not have such complete lexical databases as WordNet used in related works [5, 6], either used for bootstrapping machine learning or for evaluation.
- The nature of the Chinese language is much different from that of English which determines that the lexical-syntactic matching methods used in work [4] is hardly directly apply to Chinese language.

This paper focuses on overcoming these difficulties and exploring Chinese ontology learning methods. We make full use of the good structure of Chinese-Wikipedia and the abundant data of Baidu-Baike and Hudong-Baike, analyze the nature of the relations in the Chinese language and find relation-independent (open) methods for Chinese relation extraction, and finally achieve the Chinse ontology learning task from the online Chinese encyclopedias. We make the following contributions.

- The first system automatically learns a large-scale Chinese ontology which contains more than 5 million entities and 40 million facets. We explore the three largest Chinese online encyclopedias for ontology learning and describe how to transfer structured knowledge into ontological form.
- We analyze the nature of relations (synonymy and hyponymy) in Chinese sentences, and demonstrate that relation-independent machine learning methods are feasible for the Chinese language.
- We explain how to generate training data using the structured knowledge of encyclopedias and some general heuristics, so that the whole ontology learning process is self-supervised.
- We train some CRF machine learning models in self-supervised manner; moreover, in order to avoid the limitations of CRF models, for example, their inability to use document-level features, we also train some SVM models.

The rest of the paper is organized as follows.  Section 2 briefly reviews current studies on ontology learning.  Section 3 analyzes the ontology learning problem and gives the framework of our method. Then, we elaborate the process of learn ontological information directly from the encyclopedias in Section 4 and describe both the feasibility and the process of the self-supervised machine learning based methods in Section 5. Finally, Section 6 shows the evaluation results, and a conclusion is given in Section 7.

## 2. Related Work

Many researchers have attempted ontology learning. Here, we distinguish their works according to the source they use: text-based learning, HTML page-based learning, encyclopedia-based learning, and other source-based learning.

*Text-Based Learning*. The problem with ontology learning from text was described in detail by Buitelaar et al. [7], including methods, evaluation, and applications; their proposed ontology learning layer cake, from bottom to top, contains terms, synonyms, concepts, taxonomy, relations, and rules. Text-to-Onto [8] is an earlier ontology learning system which learns ontologies from text based on a general architecture for discovering conceptual structures. Shamsfard and Barforoush [9] proposed an automatic domain-independent ontology building approach which started from a small ontology kernel and automatically constructed the ontology through text understanding. Lee et al. [10] presented a novel episode-based ontology construction mechanism to extract domain ontology from unstructured text documents. Cimiano et al. [11] exploited Formal Concept Analysis (FCA) to acquire taxonomies from text corpus. In general, these methods relied heavily on NLP techniques, structured knowledge (e.g., dictionaries), and sometimes human interaction; when learning Chinese ontology with these methods, the learnt ontology is with low accuracy.

*HTML Page-Based Learning*. Navigli and Velardi [12] presented a tool called “OntoLearn” to learn domain ontologies from web sites; they extracted a domain terminology from web documents and arranged these terms in a hierarchical fashion. Sánchez and Moreno [13] developed an approach to automatically construct ontology from the Web starting with an initial keyword; they first used search engines to obtain web pages related to the keyword and then mined concepts from these web pages. Shinzato and Torisawa [14] provided a method to acquire hyponymy relations from HTML documents; they used itemization and listing elements in documents. These methods could only obtain shallow information as they were lacking in semantic data, and therefore could only learn some basic ontologies.

*Encyclopedia-Based Learning*. Over two million concepts with mappings to over three million unique terms were mined from Wikipedia by Gregorowicz and Kramer [3]. Ponzetto and Strube [4] derived a large-scale taxonomy from Wikipedia by labeling the semantic relations between categories. KOG [5] is an autonomous system for refining Wikipedia's InfoBox-class ontology, SVMs and Markov Logic Networks were used to solve the ontology refinement problem. YAGO [6] is a high-coverage and high-quality ontology which contains more than one million entities and five million facts; the ontology was automatically extracted from Wikipedia and unified with WordNet. YAGO2 [15] is a new version of YAGO; it enriched YAGO to 10 million entities and 447 million facts by mining temporal information and spatial information in Wikipedia. Jiang et al. [16] learnt an ontology named WorkiNet by merging Wikipedia entities into WordNet. Nastase and Strube [17] derived a large-scale multilingual concept network named “WikiNet” from Wikipedia which contains about 3.7 million concepts and 49+ million relation instances; they first utilized the existing network of categories and articles and then discovered new relations to refine the found ones. The English Wikipedia is prolific, and there are open and machine-readable standard ontologies which can be used as “gold standard,” so that good ontologies can be learnt. In contrast, Chinese-Wikipedia only has about half a million articles, and some of which only contain short descriptions; therefore, it is almost impossible to derive such large and good ontology from Chinese-Wikipedia directly.

*Other Source-Based Learning*. There are ontology learning methods based on other sources such as search engine logs [18, 19], concept maps [20], and Wiktionary [21].

As regards Chinese ontology learning, Chen et al. [22] tried to construct a core ontology from an English-Chinese bilingual term bank by using multiple translation features; their built ontologies are quite small, however, as WordNet itself suffers from low coverage. Tian et al. [23] proposed a Chinese ontology learning system based on a search engine; they investigated the importance of “verb plus noun phrase” in extracting activity concepts and successfully built a small Chinese festival ontology. The syntactic properties are too limited, in our opinion, however, and will probably lose their function in other domains. Nian et al. [24] developed a method for constructing a Chinese ontology by merging the carefully-designed structure of an expert-edited ontology called CCD and freely-edited online cyclopedias; this method, however, relies on a manually built ontology and only discussed merging entities into the ontology. Jiang et al. [25] constructed a Chinese emotion ontology from dictionaries; they first manually labeled over 7,000 affective words with affective labels and explanations as an affective lexicon, then parsed the relationships in the lexicon and built up an emotion hierarchy; the method is only focused on a special small domain and also rely heavily on human work.

The work described in [26] is the most one similar to ours; it specified a method to extract ontological information from a Chinese encyclopedia named Hudong-Baike which we will use in our work too. Our work differs in the following aspects: we will fully use the information in Hudong-Baike but [26] only used part (e.g., the redirection pages are not used), and we will also use two other Chinese encyclopedias; we will discuss relations (such as synonymy) that not included in [26]; in addition to use the structural information in encyclopedias, we also use some machine learning based methods to enrich the learnt ontology; the ontology we learnt is much larger, the scale of our ontology is in million level, while the one in [26] is in thousand level.

## 3. Problem Analysis

Buitelaar's ontology learning layer cake [7] has six levels; from bottom to top these are term extraction, synonym resolution, concept identification, taxonomy learning, relation learning, and rule learning. We will not discuss rule learning in this article, but we will describe learning instances of concepts. During our learning process, the first step is to extract candidate terms for concepts, extract terms for instances, identify synonymy relations (synonym resolution), and preliminarily distinguish concept from instance; the second step is to learn hyponymy relations and build a hierarchy of concepts and instances (taxonomy), and further distinguish concept from instance; the final step is to learn attributes of concepts and instances.

### 3.1. Chinese Corpus for Ontology Learning

In terms of English ontology construction, English Wikipedia provides a large amount of structured knowledge, and many researchers have therefore focused on ontology learning from English-Wikipedia. The structure of Wikipedia is quite good and plenty of extraction methods have been developed by earlier researchers. While Chinese-Wikipedia only contains about half a million articles, which is much less than that of the English version; nevertheless, it is still a good corpus for Chinese ontology construction, either used alone or combined with other corpora. And, fortunately, there are two other famous encyclopedias, namely Hudong-Baike and Baidu-Baike, contain a considerable amount of information like English-Wikipedia. At the time of writing the article, there are more than 4 million articles in Hudong-Baike and more than 5 million articles in Baidu-Baike. These two encyclopedias have been used by many researchers for the task of knowledge base construction [26], Linked Open Data building [27], cross-lingual knowledge linking [28], and so on.

**Figure 1:**
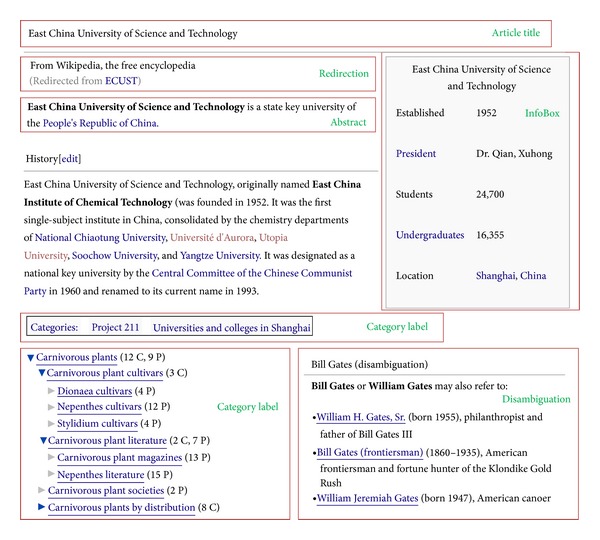
Useful structured information in encyclopedias.

Hudong-Baike and Baidu-Baike imitate Wikipedia in several aspects, and thus they also have many structured knowledge which are helpful for ontology construction. The structured information we used are article titles, article abstracts, redirection pages, disambiguation pages, category labels of articles, InfoBox modules, taxonomies, all of which are shown in Figure 1 (as the journal only accepts manuscripts in English, some examples in the paper are translated from Chinese to English), and they are also elaborated by other researchers in [29, 30]. We also mine latent relations from the text in the three encyclopedias with machine learning based methods.

### 3.2. The Encyclopedia Based Ontology Learning Method

**Figure 2:**
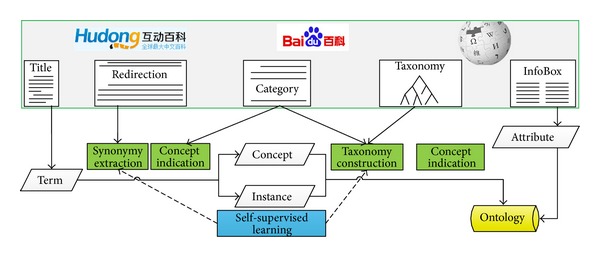
The framework of our encyclopedia-based ontology learning system.

This paper focuses on exploiting Hudong-Baike and Baidu-Baike and integrating them with Chinese-Wikipedia. We first mapping the structured data such as InfoBox modules and redirection pages into ontological information, and then using some machine learning based methods to extract more latent relations from the text of the three encyclopedias. The framework of our system is shown in Figure 2, in which the titles of articles are adopted as candidate terms for concepts and instances, the redirection pages are good clues for synonymy extraction, the category labels are chosen as candidate concepts, the category systems and taxonomies of encyclopedias are good structure for taxonomy construction (including concept-subconcept relation extraction and concept-instance relation extraction), and the InfoBox modules are useful for attribute learning. Machine learning methods are used in two processes, synonymy extraction and taxonomy construction.

## 4. Ontology Learning Method

We will elaborate the ontology learning process in detail in this section, including concept and instance extraction, synonymy relation extraction, taxonomy construction, and attribute learning.

### 4.1. Concept and Instance Extraction

All the article titles in the three encyclopedias are regarded as terms, and they are candidates for concepts and instances; the problem is how to distinguish concepts from instances. Our method is find out concepts first, and the left are instances; we determine concepts with three methods which are executed step-by-step.

- First, the category labels in the taxonomies of the three encyclopedias are adopted as concepts. Generally, the taxonomies are reviewed by administrators or high level editors and thus are highly reliable.
- Second, other category labels which not in the taxonomy systems are also good candidates for concepts; there are, however, some unreasonable categories, including (a) the empty category (the category has no subcategory or instance page): we consider the category as an instance and populate it into its parent category; (b) the category only has itself as an instance: we consider the category as an instance too, and populate it in the category's parent. After the two filtering step, the left ones are still not so reliable, and we do the following two further determinations: (1) checking the reliabilities of category labels extracted from each encyclopedia to see if category labels of any one encyclopedia are satisfactory (later experiments will show that Chinese-Wikipedia satisfy the condition); (2) checking the reliabilities of those category labels occur in two encyclopedias and those category labels occur in all the three encyclopedias, and obtaining concepts.
- Third, concepts also come from the hyponymy relation, when organizing the relations in a taxonomic manner, the entities do not at the lowest levels (leafs) are adopted as concepts.

Another fact worthy of particular attention is that a few articles have multiple meanings, which are shown by disambiguation pages; in this case, one instance is composed of the corresponding article title and the confirmed category of the title. For example, the article titled “*大学*” has a corresponding disambiguation page which shows it has four meanings: “*教育机构* (education institute, means college),” “*儒家文献古籍* (old scripture),” “*电影* (movie),” and “*杂志* (magazine).” Thus four instances, “*大学* [*教育机构*],” “*大学* [*儒家文献古籍*],” “*大学* [*电影*],” and “*大学*[*杂志*],” will be extracted.

In addition, there will always be an abstract in each article, so we provide an abstraction for each entity.

### 4.2. Synonymy Relation Extraction

As encyclopedias are generated by folk wisdom, there are many repetitive contents, either in each encyclopedia or between different encyclopedias. Therefore, we have to merge synonymous entities. For those articles with the same title in different encyclopedias we simply consider them as the same and merge them; although the method is simple, it is very helpful and very accurate. However, it is not easy to resolve synonymous articles with different titles; therefore, we provide three solutions in the article; the first one is based on the structured information (redirection page and InfoBox module) of encyclopedias; and the other two are machine learning based methods, namely the sequence labeling solution and the classification solution.

All three encyclopedias have good redirection mechanisms, and synonymous terms are redirected to the same article page. For example, there are three different Chinese terms for the term “computer”, namely, “*电脑*,” “*计算机*,” and “*电* *子* *计算机*;” the first two terms are redirected to the article titled “*电* *子* *计算机*” in Chinese-Wikipedia. The second useful structure for synonymy learning is the InfoBox module, and the fields in InfoBox modules named “*别称* (alias)” (in Baidu-Baike) and “*中文别名* (alias)” (in Hudong-Baike) list many synonyms. For example, the InfoBox of the article “*计算机* (computer)” in Baidu-Baike has the field “*别称*: *电* *子* *计算机*, *电脑*.”

The other two machine learning based solutions are quite different from traditional synonymy extraction methods, that is, our methods work in an open manner and aim at extrat as many facts as possible from the open world, while most traditional methods work in an closed manner and usually obtain relations from a given data set. We will describe them in Section 5 in detail.

### 4.3. Taxonomy Construction

Concepts should be organized to hierarchy manner (concept-subconcept relation), and instances should be populated into concepts (concept-instance relation); therefore, all the concepts and instances form an taxonomic system. The system is a directed acyclic graph (DAG) but not a tree as some nodes have more than one parents. In order to obtain the taxonomic system, we first have to extract concept-subconcept relations and concept-instance relations, and then organize these hyponymy relations as a DAG. The later step is simple (we do the work simply by identical name merging) and we only discuss the former one here; the following methods are applied.

- Transferring concept-subconcept relations from the taxonomy systems of encyclopedias. Chinese-Wikipedia and Hudong-Baike have well-structured taxonomies, from which we can extract an original taxonomy. Baidu-Baike also has its own taxonomy system, but the categories are scattered and are not organized in a strict hierarchical structure. Although Ponzetto and Strube [31] declared that “Wikipedia categories are thematically organized like a thesaurus instead of forming a fully-fledged subsumption hierarchy,” in our ontology, we consider the organization manner is reasonable. Furthermore, as the category systems are edited by category administrators or high-level editors, therefore, they are highly reliable. The problem is there are many duplicates and meaningless categories which should be filtered first, including categories that are (a) redirection categories, (b) labeled as “administration categories,” (c) generated by robots automatically but with no subcategory and with no instance page, and (d) used for mapping traditional Chinese and simplified Chinese.
- Extracting both concept-subconcept relations and concept-subconcept relations from the category systems of encyclopedias. For each article, either it is taken for a concept or an instance, there are always several category labels, which are considered as hypernyms of the current article; therefore, we can use the category system of encyclopedias to pick candidate hyponymy relations by utilizing the category labels of every article page. One problem is that not all the category labels are really reliable, thus we have to further determine the final hyponymy relations with the following two steps: (1) checking the precision of the relations extracted from each encyclopedia to see if relations of any one encyclopedia are satisfactory (later experiments will show that relations extracted from Chinese-Wikipedia meet the requirement); (2) checking the precision of those relations supported by two encyclopedias and those relations supported by all the three encyclopedias. Another problem is that most articles have more than one category labels and we have to determine the most appropriate label for each article; the following heuristics are used here: (1) if an article has only one category label, obviously it will be adopted; (2) if an article has more than one category labels and there are hyponymy relations among these labels, the ones not in the lowest level will be omitted. For example, the article titled “*郁金香* (tulip)” has category labels named “*植物* (plant)” and “*单* *子* *叶* *植物* *纲* (Monocotyledoneae).” Obviously, the article will be populated into “*单* *子* *叶* *植物* *纲* (Monocotyledoneae),” which is a descendant of “*植物* (plant).”
- Learning hyponymy relations by machine learning methods. In order to further enrich the taxonomic system, we also provide two different machine learning based solutions, a sequence labeling solution and a classification solution, to learn more hyponymy relations; both of which are similar to the synonymy learning solutions which will be described in Section 5.

### 4.4. Attribute Learning

In general OL tasks, relation learning involves three types of relations, namely, specific relation, general relation, and attribute. In this paper, we only discuss attribute learning, that is, given a concept or an instance, the aim is to find the attribute identifier as well as the value of the attribute.

**Figure 3:**
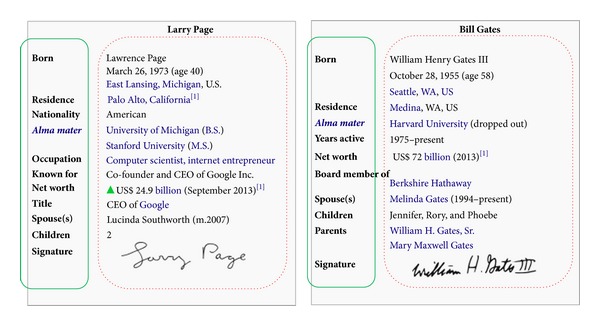
Two InfoBox examples extracted from Wikipedia, the labels in the green solid line boxes are attribute names, and the labels in the red dashed line boxes are attribute values.

The InfoBox modules of articles in all three encyclopedias are good resources for attribute extraction. In Wikipedia, an InfoBox is defined as “a fixed-format table designed to be added to the top right-hand corner of articles to consistently present a summary of some unifying aspect that the articles share and sometimes to improve navigation to other interrelated articles.” In other words, an InfoBox is a series of tabulated properties of the corresponding article.  Figure 3 shows the InfoBox modules of the article “Larry Page” and “Bill Gates.” It is very easy to parse them into the attributes of entities.

## 5. Self-Supervised Relation Learning

In the ontology learning process described in this article, three types of relation are involved: synonymys, hyponymys and attributes. For attribute learning, we only use a method based on the structured data in encyclopedias and we have elaborate it Section 4.4. Here, we will focus on discussing the solutions of the first two relations.

In traditional machine learning based relation extraction methods, there should be a specified relation and many labeled examples (both positive and negative) of the relation; that is, they work in supervised manner. Take synonym relation extraction for example, the traditional methods need to know the problem words (the words that specify the synonym relation) and the target words (the words to determine whether they are synonyms of the problem words or not) first and then identifying synonyms using supervised machine learning methods. The methods violate our large-scale onlogical information extraction in three aspects: the number of extractions is limited, they can only extract synonyms from a set of predetermined words, and they need the manual building of training corpus. Therefore, we have to find an automatic (or open) method, which could doing the extraction task in an unsupervised or semisupervised manner, but is it possible? In this section, we will analyze the nature of relations (synonymy and hyponymy) in Chinese and proof that self-supervised learning is applicable and then elaborate two kinds of self-supervised learning models for synonymy relation learning.

### 5.1. The Nature of Synonymys and Hyponymys in Chinese

Open Information Extraction [32] and Hearst's work [33] have shown that relations of entities are consistently expressed by a compact set of relation-independent lexicon-syntactic patterns. Although no previous work has demonstrated that the same could apply in Chinese, we believe that there are some similar patterns. In order to proof our hypothesis, we studied a number of sentences which contain synonymys and hyponymys to see if there are any lexicon-syntactic patterns which cover most of the studied sample instances. Take synonym relation for example, first, we extracted 100 pairs of synonymous words from the redirection pages of encyclopedias (in order to ensure validity, all these synonym pairs are supported by two or three of the encyclopedias); then, for each pair of synonyms, we extracted 10 different sentences that contained the two synonymous words; finally, we summarized patterns from these 1000 sentences. The lexicon-syntactic patterns derived are shown in Table 1 (*E*_*n*_ stands for an candidate synonymous word), we discovered nearly one hundred patterns and some of those that occurred most frequently are listed.

In our sample sentences, almost every pattern occurs multiple times and expresses more than one pair of synonyms. When the contextual words around one pair of candidate words matches a pattern that indicates synonyms, then the two entities are probably synonyms. In other words, whether two entities are synonyms is determined by the contextual words around them but not the entities themselves. This suggests that open methods for synonym extraction are feasible and that learning more patterns will obtain more synonyms.

The proof process for open hyponymy relation is similar to that of the open synonymy extraction, thus we only show the obtained lexicon-syntactic patterns in Table 2. Now, we are going to learn some models for synonymy extraction and hyponymy extraction.

### 5.2. Sequence Labeling Solution for Synonymy Extraction

*Sequence Labeling Problem*. In machine learning, sequence labeling involves the algorithmic assignment of a categorical label for each member of a sequence of observed values; the input *X* is a sequence of observations, and the output *Y* represents hidden sequential states that need to be inferred from the observations; all the output *y*_*i*_ form a chain with an edge between each *y*_*i*−1_ and *y*_*i*_, which means they follow the first-order Markov assumption. Commonly used sequence labeling models are the Hidden Markov Model (HMM), Maximum Entropy Markov Model (MEMM) and Conditional Random Field (CRF). CRF [34] is a discriminative undirected probabilistic graphical model which is used to encode known relationships between observations and construct consistent interpretations. It is often used for labeling sequential data such as natural language text, and its applications include word segmentation, part-of-speech tagging, named entity recognition, and relation extraction.

**Figure 4:**
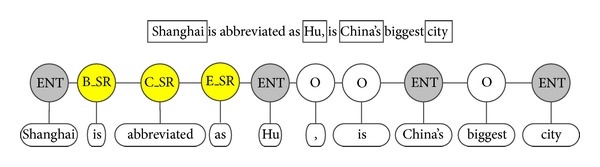
A labeled example of SR-CRF.

*Modeling*. In synonymy learning here, the observable variables *X* are Chinese word sequences, and hidden states *Y* are tags defined by us which identify entities, relations and other words in *X*; we assume *Y* satisfied with the first-order Markov assumption about dependencies among the output states. Therefore, we can model the task as a sequence labeling problem. We train a CRF model using PocketCRF (downloadable free from http://code.google.com/p/pocketcrf/) and name it SR-CRF. Entities (concepts or instances) are already extracted, and will be labeled as *ENT*. Those pairs of adjacent entities (no other entity between them) with certain distance (e.g., no more than five words) are candidate pairs for synonyms and the surrounding context of each pair of entities is seen as potential evidence for synonym relation. These contextual words will be assigned the following labels: *B*_*SR*, the opening word of the synonym relation; *C*_*SR*, the center word of the synonym relation; *E*_*SR*, the end of the synonym relation; *O*, words that do not express a synonym relation.  Figure 4 shows a labeled example, which is used to identify the synonym relation “is abbreviated as” of two entities, “Shanghai” and “Hu.” The words of another two adjacent pairs of entities are labeled *O* as they are not synonyms.

*Feature Selection*. Jiang and Zhai [35] showed that, for relation extraction, basic unit features were sufficient to achieve satisfactory performance, and over-inclusion of complex features can damage performance. Though their experiments treated relation extraction in English, we believe that they are also applicable to Chinese relation extraction. Thus, in this article, we do not use any deep-NLP features and only do the word segmentation preprocess by ctbparser, (downloadable free from http://sourceforge.net/projects/ctbparser/). The features we use are “*W*_−2_,” “*W*_−1_,” “*W*_0_,” “*W*_1_,” “*W*_2_,” “*W*_−1_ *W*_0_,” and “*W*_0_ *W*_1_,” where index 0 indicates the current word in focus and indices −*n*/*n* indicate the *n*th left/right word of the current word.

*Self-Supervised Training*. The most important advantage of SR-CRF and the other models mentioned in the paper is that they generate training examples automatically, that is, they are self-supervised. We use the synonyms which have been extracted from encyclopedias and some relation-independent heuristic rules to generate training data. The candidate sentences for training data are sentences which contain at least one pair of candidate entities. The positive example sentence is determined by the heuristic rule “the pair of candidate entities are synonyms (already extracted in Section 4.2);” in order to ensure validity, here we choose the synonym pairs supported by at least two encyclopedias. While the heuristic rules for generating negative examples are more complicated and we have to leverage the structured information in encyclopedias to determine those pair of entities that are not synonyms. For entity pairs that are not synonyms, we use the heuristic rules like; the two entities are of different types in Wikipedia, for example, one corresponds to a category label and the other is an instance page of the category; the two entities are of different domain in the category system of Wikipedia, for example, one is a plant and the other is an animal. Take the entity pairs in Figure 4 for example, there are two pairs of adjacent entities that are not considered as synonyms; for the first pair of entities, “Hu” and “China,” the latter is a category label of the former; and for “China” and “city,” the first is a country but the second is not a country.

The automatically labeled training examples are formatted as required and then the CRF learner takes them as input to train the synonym extraction models. The training process is time-consuming and it will usually continue for several hours to several days.

*Synonymy Extraction*. For other input sentences that satisfy the requirement of candidate sentences for training data, by initially performing word segmentation, candidate entities are also determined by the rule “the pairs of adjacent entities with certain distance.” Then, SR-CRF makes a single pass over them and labels the contextual words between each candidate pair of entities. If the contextual words of one pair of adjacent entities are labeled with synonym tags, then the two entities will probably be selected as synonyms. We also record in detail how many sentences and how many distinct patterns support every pair of synonymous words; this supporting information is important in making the trade-off between precision and recall.

### 5.3. Classification Solution for Synonymy Learning

Similarly to other systems which model extraction from natural language text as a sequence labeling problem, the sequence labeling solution has several limitations; for example, it cannot exploit document-level features, and the two target entities must occur in the same sentence. In order to compensate for these limitations, we also model relation learning problems as classification problems.

The relation learning tasks in this article are typical classification problems; for example, in synonym relation learning two entities are either synonyms or they are not. For every target entity, there is always a corresponding article page which contains richer information for relation learning.

The classification model we use is Support Vector Machine (SVM) [36]. SVM is a well-known nonprobabilistic binary linear classifier which has been successfully used in natural language processing and text categorization. For synonym learning, we train a SVM model named SR-SVM using lib-SVM (downloadable free from http://www.csie.ntu.edu.tw/~cjlin/libsvm/). The training examples are also generated automatically with the same method as SR-CRF, and more than ten features are adopted, the most important four are as follows.

- Cosine similarity of the corresponding articles: articles which describe synonymous entities are always with high similarity.
- Edit distance (Levenshtein distance (http://en.wikipedia.org/wiki/Levenshtein_distance)) of the two entities: synonymous entities always share many words or characters, thus their edit distance is small.
- Cooccurrence probability in article level: in general, synonymous entities cooccur often. In article level means the number of occurrences takes artitle as unit.
- Co-occurrence probability in sentence level: similarly, sentence level means the number of occurrences takes sentence as unit.

The training examples are formatted as required and lib-SVM will train the classification model, the training process is much more efficient than the training process of SR-CRF.

Intuitively, the candidate target entity pairs for classification are arbitrary entity pairs, but it will generate about 25 trillion pairs (square of the total number of articles), which is unreasonable. In order to reduce the number of entity pair, we make the assumption that “if two entities do not co-occur in their corresponding articles, they are probably not synonyms.” To verify our assumption, we have manually extracted 100 synonymous entity pairs from a Chinese dictionary which are not indicated by the redirection pages and InfoBox modules of encyclopedias, and have got the result that 98 pairs follow our assumption. Therefore, for each entity, we only generate entity pairs with those entities occur in the corresponding article of the entity. For every chosen pair, SR-SVM will predicate whether they are synonyms or not.

### 5.4. Machine Learning Based Solutions for Hyponymy Extraction

Similarly, we also train a sequence labeling model and a classification model for hyponymy relation extraction and name them HY-CRF and HY-SVM, respectively; the training and extraction processes are similar to those of the synonymy learning models, and we do not retell them here.

## 6. Experiment and Evaluation

In this section, we first describe the experiment data used for ontology learning; then we show the learnt ontology in detail and evaluate the precision of each element of the ontology; finally, we compare the learnt ontology with other famous ontologies in scale. In general, there are four types of methods for ontology evaluation: the “gold standard” ontology-based method, the application-based method, the data-based method (which compares the ontology with domain data and judges its scale), and the domain expert-based method. As there is no “gold standard” ontology in Chinese and we have not developed any application based on our ontology yet, we will evaluate the learnt ontology with two other methods, assessing its precision manually and measuring its scale by comparing it with other ontologies.

### 6.1. Experiment Data

In order to use the online encyclopedias, we have to download articles from them first. It is easy to download articles from Chinese-Wikipedia as it provides a dump address (http://download.wikipedia.com/zhwiki/), but the other two encyclopedias have no such service, and therefore we have to develop crawlers to derive their contents. We have successfully crawled 4,729,672 articles from Baidu-Baike and 3,680,773 articles from Hudong-Baike. Detailed information about the three encyclopedias is provided in Table 3 (the version of the Chinese-Wikipedia we used is dated as 2012-12-10, and the versions of the other two encyclopedias are around December 2012). The numbers of crawled (downloaded) articles are less than the official numbers of the encyclopedias, we have discovered two reasons for this: there are some isolate articles which cannot be found by our crawlers, and some articles are protected which prohibit crawlers to crawl. The table also lists the quantities of structured information.

### 6.2. Ontology Learning Result Overview

First, we give the final leant ontology in Table 4. We learn an ontology with 264,940 concepts, 4,902,180 instances, 812,239 synonymy relations, 711,232 concept-subconcept relations, 25,960,023 concept-instance relations, 6,926,942 attribute facts, and, in total, 39,577,556 facts. We name the learnt ontology as SSCO and published it on the Web (http://ssco.zhishimofang.com).

### 6.3. Detail Result and Evaluation

It is very difficult to evaluate the precision of SSCO. Even when evaluating it manually, it is not easy to judge whether some facts are correct or not. We request 5 graduate students of our laboratory to evaluate our ontology; for each fact, if 4 or 5 of them consider it correct, we determine the fact as correct, otherwise as false; for each element type, we randomly select 500 examples and ask them to determine.

*Concept*. Generally speaking, it is meaningless to say that a concept is incorrect; here we say a concept is incorrect when “taking an instance as a concept.” First, 104,803 concepts obtained from the taxonomies of Hudong-Baike and Chinese-Wikipedia, with precision of nearly 1.0; the high precision is benefited from the good taxonomy structure of the two encyclopedias.

Second, for the category labels not in the taxonomies, we first filter the illegal concepts (described in Section 4.1) and then divide them into three reliable levels: the ones occur in only one encyclopedia, the ones occur in two of the encyclopedias, and the ones occur in all the three encyclopedias. In the first level, we find that extractions from Chinese-Wikipedia have precision about 0.990, thus all these extractions are considered correct directly; while the extractions only supported by Hudong-Baike and only supported by Baidu-Baike only have precision of 0.824 and 0.672 respectively, and we further evaluate those extractions supported by both of them (the second level) and get precision of 0.972. The quantities of extracted concepts and the corresponding precision are listed in Table 5. In the aspect of quantity, there are only 7,622 category labels in Chinese-Wikipedia, because most of the category labels of Chinese-Wikipedia are organized in its own taxonomy; while the other two encyclopedias both have a lot of category labels which are not in their taxonomy systems. In the aspect of precision, the extractions only from Hudong-Baike or Baidu-Baike are not so reliable and we have to find other evidence for them. In total, in this step, we got 84,545 concepts.

Third, other 75,592 concepts are obtained from the hyponymy relations, with precision of 0.964.

Finally, 264,940 concepts are obtained, with average precision of 0.981.

*Instance*. The category labels and article titles that not adopted as concepts are instances, 4,902,180 in total. The precision of instances is really meaningless, thus we do not have to do the evaluation.

**Figure 5:**
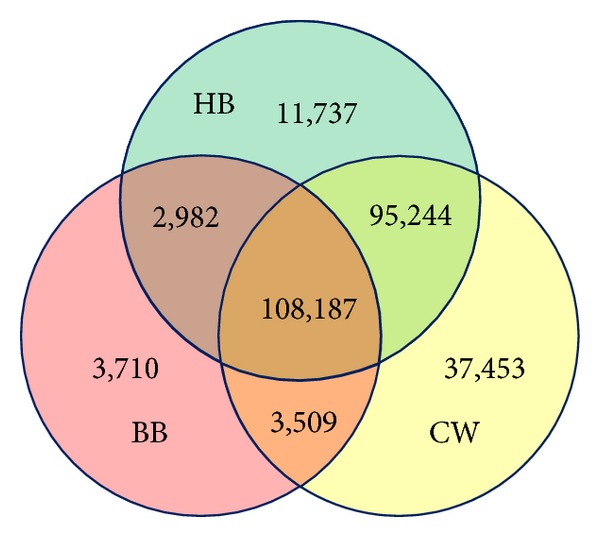
The synonym relations extracted from the redirection pages and InfoBox modules of encyclopedias.

*Synonymy Relation*. The first part of synonymy relations are extracted from the redirection pages and the InfoBox modules of the encyclopedias; Figure 5 shows the detailed result, and we totally get 262,822 synonymy relations; as the redirection pages and the InfoBox modules are also very reliable, thus the precision for the corresponding synonym relations is good (nearly 1.0).

We also apply two kinds of machine learning methods to obtain synonym relations. Precision and recall are commonly used metrics in machine learning based information extraction and therefore, we adopt them to evaluate our system. The precision at a specific threshold th is defined as the number of correct extractions divided by the total number of extractions at or above a threshold th (in SR-CRF and HY-CRF, th means how many sentences are sufficient to support the synonymy relation between the two entities, and in SR-SVM and HY-SVM, th means the probability given by the models). The recall at a threshold th is the number of correct extractions at or above th divided by the total number of correct extractions at all thresholds. Note that the recall is with respect to all the correct results that our system extracted but not all the potentially unknown correct results in the total corpus or the whole web. This is consistent with the recall metric used in TREC (Text REtrieval Conference): only count correct instances that are in the data collection actually processed by a system. As with most other information extraction systems, our system has to make trade-off between precision and recall. By raising the threshold that deems a synonymy relation true, we increase precision and decrease recall, whereas lowering the threshold has the opposite effect.

**Figure 6:**
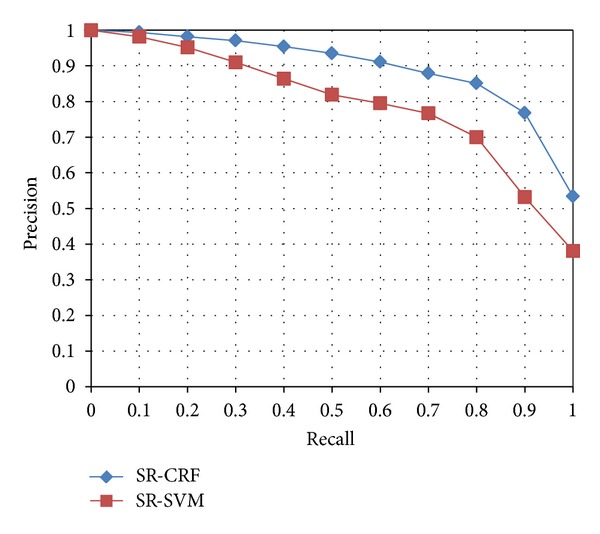
Precision-recall curves of SR-CRF and SR-SVM.

It is unreasonable to evaluate all the results extracted and thus, we randomly select a set of sample synonymy relations from the result set, and ask the 5 graduate students to evaluate them. The precision-recall curves of the two models are shown in Figure 6. In our ontology learning process, the precision is important; in the article we chose the lower limit of 0.9. In this limitation, in the SR-CRF extraction process, we conclude that 3 sentences are sufficient to determine a synonym relation true and obtain 357,009 pairs of synonyms with precision of 0.912; meanwhile, SR-SVM bring us 80,182 pairs of synonyms with precision of 0.9. For the remaining extractions, we choose those pairs are extracted by both models for further evaluation, and we obtain another 112,226 synonym pairs with precision of 0.932.

In total, we get 812,239 synonymy relations with average precision of 0.943 and show them in Table 6.

*Taxonomy Learning and Concept-Instance Relation Learning*. By mining the taxonomy systems of Hudong-Baike and Chinese-Wikipedia, we obtain 442,894 concept-subconcept relations among 104,803 concepts; as the taxonomy systems of the two encyclopedias are highly reliable, thus the precision of these extraction are nearly 1.0.

Meanwhile, a large number of hyponymy relations are obtained by collecting the category labels of articles. Similarly, we also divide these extraction into three reliable levels, extractions supported by only one encyclopedia, by two encyclopedias, and by all the three. In the first level, only Chinese-Wikipedia performs well once again and bring us 1,549,103 relations, with precision of 0.978. While evaluating those extractions supported by both Hudong-Baike and Baidu-Baike, we get acceptable precision of 0.944 as well and obtain 13,902,238 relations in total.

And two machine learning methods are applied to extract the hyponymy relations too; the trade-off decisions between recall and precision are almost the same with those of the synonymy relation extraction machine learning methods. In summary, HY-CRF return 21,820,220 relations of which 9,723,819 ones are new; the precision is 0.916; HY-SVM return 2,129,348 hyponymy relations of 723,076 ones are new, with precision of 0.9; by combining the left extractions of HY-CRF and HY-SVM, we again get 330,125 new hyponymy relations with precision 0.928. Therefore, we totally obtain 26,671,255 hyponymy relations with precision of 0.935. The results are described in Table 7.

Now, we are going to divide the relations into concept-subconcept relations and concept-instance relations, the dividing principle is simple, in the final taxonomic system, those relations with leaf nodes (nodes that have no subnodes) are concept-instance relations, and others are concept-subconcept relations. Finally, there are 711,232 concept-subconcept relations and 25,960,023 concept-instance relations.

*Attribute*. The attributes are all extracted from the InfoBox modules of the three encyclopedias, 6,926,942 in total; and because the InfoBox modules are with good reliability, thus the precision of these extractions are nearly 1.0 too.

### 6.4. Comparison of SSCO with Other Ontologies

It is unfair to compare the size of SSCO with that of other existing ontologies directly as the structures of different ontologies are different. For example, some ontologies largely consist of entities but other ontologies largely contain relation triples. Therefore, we simply list several well-known ontologies in Table 8. From the aspect of structure, SSCO has abundant ontological information element such as concept, instance, taxonomy, synonymy, and attribute, while most other listed ontologies only have entities and general relations between entities. From the aspect of scale, although SSCO is not the largest one, its scale is at the same level as the largest one named YAGO2 and is much larger than others. Moreover, in Chinese ontologies, our SSCO is much larger than HowNet (the most well-known open Chinese knowledge base) and the ontology learnt in work [26].

## 7. Conclusion and Future Work

This paper focuses on Chinese ontology learning from online encyclopedias. Apart from Wikipedia, we also exploit other two of the largest online Chinese encyclopedias, namely, Hudong-Baike and Baidu-Baike, for ontology learning. First, we use the terms which appear as category labels of encyclopedias as candidate concepts and titles of normal articles as candidate instances and discover synonymy relations. Second, we extract an original taxonomy from the taxonomy systems of Hudong-Baike and Chinese-Wikipedia, and mine concept-subconcept relations and concept-instance relations from the category systems of encyclopedias. Finally, we extract attributes of concepts and instances from the InfoBox modules. Meanwhile, in order to learn more ontological relations, we apply two kinds of machine learning based methods for synonymy relation extraction and hyponymy relation extraction; all these methods are self-supervised as the training examples are automatically generated from the structured knowledge of encyclopedias with some general heuristic rules.

We learn a Chinese ontology named SSCO, which contains 264,940 concepts, 4,902,180 instances, 812,239 synonymy relations, 711,232 concept-subconcept relations, 25,960,023 concept-instance relations, 6,826,942 attribute facts, and, in total, 39,577,556 facts. When evaluate its precision manually and compare it with other ontologies, SSCO is satisfactory in both precision and scale.

Future works will focus on enriching SSCO in each element, especially in attribute learning; we are also going to link SSCO with DBPedia (http://dbpedia.org/About), the most famous open structured knowledge base.

## Figures and Tables

**Table 1:** Lexicon-syntactic patterns of synonyms in the sample sentences.

Pattern	Example
*E*_1_ is commonly called as *E*_2_	Jisuanji (computer) is commonly called as Diannao
*E*_1_ is abbreviated as *E*_2_	Shanghai is abbreviated as Hu
*E*_1_ is also named as *E*_2_	Hehua (lotus) is also named as Lianhua
*E*_1_ is also called as *E*_2_	Oceania is also called as Australia
*E*_1_ is originally called as *E*_2_	Laoshe is originally called as Shu Qingchun
*E*_1_ is anciently named as *E*_2_	Xi'an is anciently named as Chang'an
*E*_1_ is *E*_2_ *ߣ*s synonym	Like is love's synonym
*E*_1_ is *E*_2_ *ߣ*s abbreviation	Hu is Shanghai's abbreviation

**Table 2:** Lexicon-syntactic patterns of hyponymys in the sample sentences.

Pattern	Example
*E*_1_ is a type of *E*_2_	Tablet is a type of computer
*E*_1_ is an *E*_2_	China is a country
*E*_1_ is a kind of *E*_2_	Human is a kind of mammal
*E*_1_ such as *E*_2_ and *E*_3_	Animals such as lion and tiger
*E*_1_ there is/are *E*_2_, *E*_3_, and so on	Dynasties there are Han, Tang, and so on
*E*_1_ is one of *E*_2_	China is a developing country

**Table 3:** Information crawled from online encyclopedias.

	Article	Category	InfoBox	Redirection
Baidu-Baike	4,729,672	635,424	171,532	150,003
Hudong-Baike	3,680,773	213,176	641,251	258,720
Chinese-Wikipedia	462,653	107,233	74,293	322,922

**Table 4:** The detail size of each element of SSCO; the hyponymy relations are divided into concept-subconcept relations and concept-instance relations.

Element	Size
Concept	264,940
Instance	4,902,180
Synonym relation	812,239
Concept-subconcept relation	711,232
Concept-instance relation	25,960,023
Attributes	6,926,942

Total facts	39,577,556

**Table 5:** Concept extraction result.

Concept source	Quantity	Precision	Opt
From toxonomy system	104,803	1.000	Select
From category label			
In Chinese-Wikipedia	7,622	0.990	Select
In Hudong-Baike	82,328	0.824	Discard
In Baidu-Baike	430,951	0.672	Discard
In Hudong-Baike and Baidu-Baike	76,923	0.972	Select
Total	84,545		
From hyponymy relation	75,592	0.964	Select

Total	264,940	0.981	

**Table 6:** The detail results of synonymy extraction.

Relation source	Quantity	Precision
Redirection page and InfoBox module	262,822	1.000
SR-CRF	357,009	0.912
SR-SVM	80,182	0.900
The remaining ones in both SR-CRF and SR-SVM	112,226	0.932

Total	812,239	0.943

**Table 7:** Hyponymy relation extraction result.

Relation source	Quantity	Precision
From toxonomy system	442,894	1.000
From category label		
In Chinese-Wikipedia	1,549,103	0.978
In Hudong-Baike and Baidu-Baike	13,902,238	0.944
From HY-CRF	9,723,819	0.916
From HY-SVM	723,076	0.900
From the remaining of HY-CRF and HY-SVM	330,125	0.928

Total	26,671,255	0.935

**Table 8:** Size of other ontologies.

Ontology	Entities	Facts
KnowItNow	N/A	25,860
KnowItAll	N/A	29,835
SUMO	20,000	60,000
OpenCyc	47,000	306,000
Cyc	500,000	5,000,000
WordNet	155,287	479,887
YAGO	1,056,638	5,000,000
YAGO2	9,800,000	120,000,000
HowNet	191,924	462,433
CCD	66,590	N/A
CCE	2,000,000	N/A
The ontology in [26]	822,135	5,237,520
